# Albumin-Bilirubin Grade as a Novel Predictor of Survival in Advanced Extrahepatic Cholangiocarcinoma

**DOI:** 10.1155/2018/8902146

**Published:** 2018-12-02

**Authors:** Yong Wang, Qing Pang, Hao Jin, Lei Zhou, Xiaosi Hu, Zhen Qian, Zhongran Man, Song Yang, Huichun Liu

**Affiliations:** Department of Hepatobiliary Surgery, The First Affiliated Hospital of Bengbu Medical College, 233000 Bengbu, Anhui, China

## Abstract

**Aim:**

Child-Pugh (CP) grade has been used to assess liver function and postoperative outcomes in biliary tract neoplasms. The aim of this study was to preliminarily explore the prognostic significance of an alternative model of liver function, called albumin-bilirubin (ALBI) grade, in patients with extrahepatic cholangiocarcinoma (EHC).

**Methods:**

A total of 109 advanced EHC patients, who received percutaneous transhepatic biliary stenting combined with iodine-125 seed implantation from January 2012 to April 2017 in our department, were enrolled. Preoperative clinical data were collected to calculate the CP and ALBI grades. The performance of ALBI score in predicting postoperative death was compared with that of CP score by using the receiver operating characteristic (ROC) curve. Kaplan-Meier analysis and Cox regression model were performed for overall survival (OS) analysis.

**Results:**

The median survival time of our cohort was 12 months, and the 1-year and 2-year survival rates were 56.9% and 12.8%, respectively. The area under the ROC curve of ALBI score for predicting death was significantly greater than the CP score (0.751, 95% CI: 0.641–0.861, *P* < 0.001 vs. 0.688, 95% CI: 0.567–0.809, *P* < 0.001). The univariate analysis revealed that the factors related to overall survival of EHC were carbohydrate antigen 19-9, total bilirubin, albumin, ALBI grade, and CP score. In multivariate analysis, ALBI grade (HR = 1.65, 95% CI: 1.04–2.61, *P* = 0.032), but not CP score, was identified as an independent prognostic model.

**Conclusion:**

We demonstrated that the ALBI grade could be used as a predictor of survival in unresectable EHC patients.

## 1. Introduction

Cholangiocarcinoma (CCA) represents a diverse group of aggressive malignancies arising from the damaged biliary epithelial cells. It accounts for approximately 10%–25% of all hepatobiliary malignancies and affects mainly the elderly population. Over the past several decades, the incidence of CCA has progressively increased worldwide [1]. According to the tumor location, CAA can be classified into intrahepatic cholangiocarcinoma (IHC) and extrahepatic cholangiocarcinoma (EHC). The latter type consists of hilar cholangiocarcinoma (HCCA) and distal cholangiocarcinoma (DCCA) and accounts for about 80% of cases [1]. Currently, due to the difficulty in the early diagnosis and the limited treatments, the prognosis of EHC remains poor, with a 5-year overall survival (OS) rate of 5–17% [2]. To date, simple models to assess EHC prognosis are too limited. Therefore, the exploration of new prognostic models, preferably those simply obtained from serum markers, is important.

Currently, surgical resection with negative margins remains the primary radical approach for early EHC [3], while only a small number of patients are candidates for surgery due to the advanced stage of presentation [4]. Chemotherapy is the primary treatment of choice for unresectable EHC while the effectiveness is unsatisfactory [4]. Recently, the approaches of palliative biliary drainage and biliary stent placement have been used to relieve biliary obstruction in EHC. Due to the rapid ingrowth of tumor, however, the biliary stent alone could not yield a satisfactory treatment effect [5]. Since 2012, we have routinely used the combined treatment of percutaneous transhepatic biliary stenting (PTBS) with iodine-125 (^125^I) seed intracavitary irradiation in advanced EHC, and it significantly prolongs stent patency time and improves survival compared with biliary stent alone [6].

A growing body of research suggests that several preoperative indicators of liver function, albumin (ALB), and total bilirubin (TBIL), in particular, can serve as valuable predictors of survival in EHC. As a recognized model for evaluating liver function, the Child-Pugh (CP) grade has been widely used to estimate the prognosis of hepatic cirrhosis and hepatocellular carcinoma (HCC) [7]. Moreover, CP grade is also significantly correlated with survival in other neoplasms, including EHC [8, 9]. Hyun et al. recently reported that, in EHC patients who received biliary stenting, CP grade seemed to be one of the important factors affecting survival [10]. However, as the high incidence of obstructive jaundice in EHC, advanced stage in particular [11, 12], the majority of patients belong to advanced CP grade. Moreover, in case of EHC, ascites and encephalopathy can be due to tumor spread and cholangitis. Thus, CP grade may not be very appropriate to evaluate the prognosis in EHC. Recently, a novel evaluation model for liver function, called albumin-bilirubin (ALBI) grade, has been proposed [13]. This simple model is calculated by using only ALB and TBIL. Recent studies have reported that the ALBI grade has a better performance of evaluating liver function, complication, and prognosis than CP grade in liver diseases [14, 15]. However, to our knowledge, ALBI grade has never been investigated in EHC patients, and here, we preliminarily explored the prognostic significance of ALBI in advanced EHC.

## 2. Methods

### 2.1. Patients

The medical records of advanced EHC patients, who received PTBS combined with ^125^I seed implantation from January 2012 to April 2017 in our department, were retrospectively analyzed. The inclusion criteria were as follows: (1) either HCCA or DCCA with histological confirmation or clinical diagnosis; (2) unresectability or refusal surgery; and (3) received PTBS combined with ^125^I seed implantation. Exclusion criteria were as follows: (1) benign bile duct stricture; (2) coexistent hepatic diseases that greatly influence liver function; (3) a history of bile duct surgery, systematic chemotherapy, or other curative treatments for EHC; (4) IHC; and (5) incomplete clinical or follow-up information. Finally, 109 patients met the above criteria. This report was abided by the transparent reporting of a multivariable prediction model for individual prognosis or diagnosis (TRIPOD) [16]. Our study was in compliance with the Declaration of Helsinki [17] and was approved by the ethics committee of our hospital.

### 2.2. Surgical Procedures

Percutaneous transhepatic cholangial drainage was performed under the guidance of ultrasound in advance. About one week later, we performed the PTBS and ^125^I seed implantation under digital subtraction angiography. The process of PTBS has been described previously [11]. The embedded number of ^125^I particles was calculated as follows: (length + width + height of tumor) (in cm)/3 × 5 ÷ activity per particle (in mCi). The ^125^I particles were separated with the average spacing of 0.6–1.0 cm. Under the guidance of a guidewire, the “P”-type tube containing ^125^I particles was inserted into the cavity of biliary stent.

### 2.3. Data Collection and Follow-Up

The electronic medical records were used to extract the following information: age; gender; tumor location; preoperative indicators of liver function (alanine aminotransferase (ALT), aspartate aminotransferase (AST), alkaline phosphatase (ALP), TBIL, direct bilirubin (DBIL), gamma-glutamyl transpeptidase (GGT), prothrombin time (PT), international normalized ratio (INR), and ALB); ascites status; degree of hepatic encephalopathy; C-reactive protein (CRP); carbohydrate antigen 19-9 (CA19-9); and carcinoembryonic antigen (CEA). The CP grade was evaluated by using five variables, including TBIL, ALB, PT, ascites, and encephalopathy. ALBI score is 0.66 × log_10_ TBIL (*μ*mol/l)-0.085 × ALB (g/l), and ALBI grade was defined by the resulting score (grade 1: ≤−2.60; grade 2: −1.39 to −2.60; grade 3: >−1.39) [13].

All patients were routinely followed up in the outpatient department until September 2017 or death. The follow-up information included routine biochemical test, tumor markers, and imaging evaluation.

### 2.4. Statistical Analysis

SPSS version 21.0 (IBM Corp., USA) was used to analyze the clinical data. Continuous variables with normal distribution were expressed as mean ± standard and those with nonnormal distribution were expressed as median (range). Differences between subgroups were compared by using *t*-test, Wilcoxon's test, or *χ* ^2^ test, as appropriate. The optimal cutoff value of continuous variable is determined by the largest value of Youden's index (sensitivity + specificity − 1). The area under the receiver operating characteristic (ROC) curve was used to measure the performance of ALBI and CP scores in predicting postoperative death.

OS was the primary outcome and was estimated by the Kaplan-Meier curve with log-rank test. All the single variables that were significant or nearly significant (*P* < 0.10) in the univariate analysis entered into the multivariate analysis model. Then, the ALBI grade entered into the above multivariate analysis model one by one to adjust these single factors (removal of the factors contained in ALBI score). It was considered statistically significant if *P* < 0.05.

## 3. Results

### 3.1. Characteristics of Patients and Assessment of Efficacy

All the 109 EHC patients were successfully implanted with biliary stent and ^125^I particles. The baseline data of these patients were presented in Table 1. 70 (64%) cases were clinically or pathologically diagnosed as HCCA and 39 (36%) cases were DCCA. There were 71 (65%) men and the mean age was 68.9 ± 11.1 years.

According to the CP grade, the majority of patients were stratified into grade B (*n* = 106, 97%). According to the ALBI grade, there were 62 (57%) patients with grade 3, 46 (42%) patients with grade 2, and 1 patient with grade 1.

Symptoms such as jaundice, pruritus, and fever were significantly relieved in different degrees after the operation. During the hospitalization, 15 out of the 109 patients (14%) had postoperative complications. 10 cases of hyperamylasemia were cured after the application of somatostatin and symptomatic treatment. Five cases of biliary tract infection were controlled and discharged after opening the “P”-type tube drainage and 3–7 days of antibiotic treatment. The 30-day postoperative mortality was 0.9% (1 patient died from recurrent biliary tract infection and multiple organ dysfunction syndrome at 28 days after operation). No other patient died within 90 days postoperatively.

### 3.2. Associations between ALBI Grade and Clinical Variables

**Figure 1:**
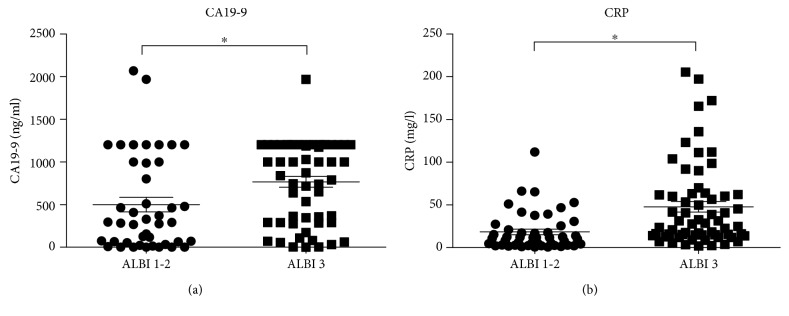
Scatter plots revealing the associations between ALBI grade and CA19-9 (a) and CRP (b) (^∗^ *P* < 0.05).

Demographic information, serologic tests, and tumor location of patients stratified by the ALBI grade are shown in Table 1. Of these clinical variables, CA19-9, ALT, TBIL, DBIL, ALB, CRP, PT, PT-INR, and CP score were significantly different between patients with ALBI grades 1–2 and that with ALBI grade 3. The scatter plots further revealed the positive associations between ALBI grade and CA19-9 (Figure 1(a)), CRP (Figure 1(b)).

### 3.3. Predictive Potentials of ALBI vs. CP Scores for Detecting Death

**Figure 2:**
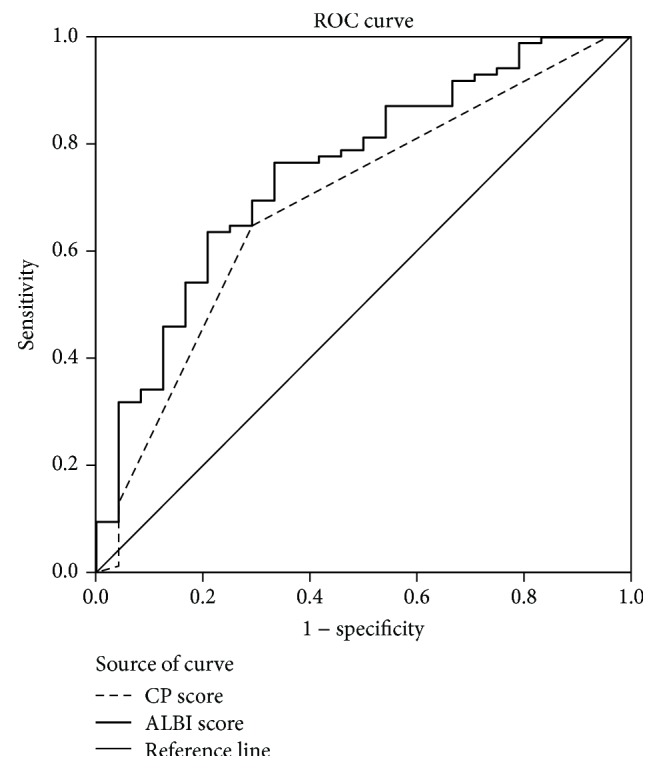
Predictive potentials of CP vs. ALBI scores for detecting postoperative death.

The areas under the ROC curve for the ALBI and CP scores were 0.751 (95% CI: 0.641–0.861, *P* < 0.001) and 0.688 (95% CI: 0.567–0.809, *P* < 0.001), respectively, for predicting postoperative death. As shown in Figure 2, compared with CP score, the ALBI score showed a higher accuracy in detecting death.

### 3.4. Predictors of OS

**Figure 3:**
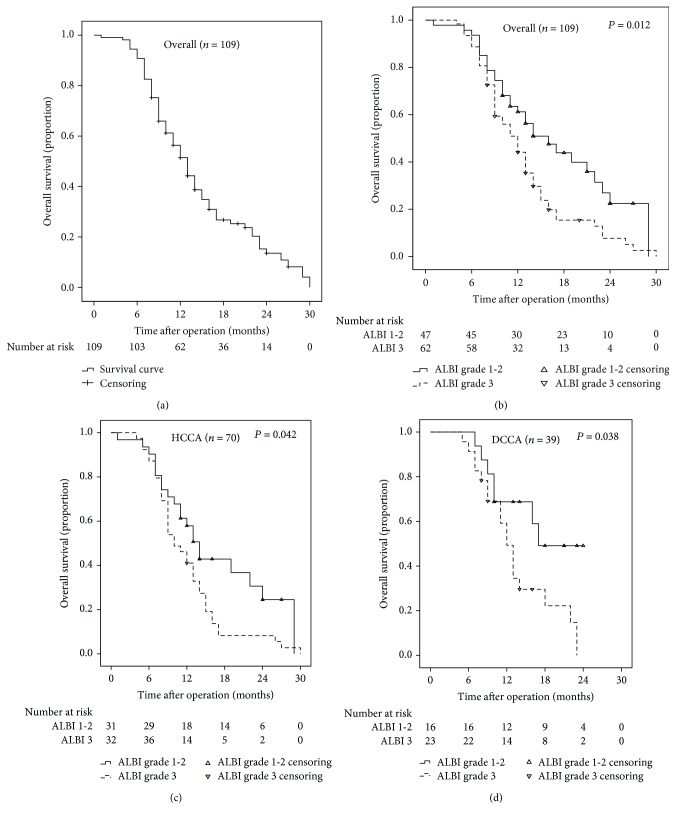
The survival curves of unresectable EHC patients. The whole cohort (a), stratified according to ALBI grade in the whole cohort (b), stratified according to ALBI grade in the HCCA subgroup (c), and stratified according to ALBI grade in the DCCA subgroup (d).

Figure 3(a) showed the Kaplan-Meier curve of cumulative OS in the whole cohort. In general, the median survival time was 12 months, and the 1-year and 2-year OS rates were 56.9% and 12.8%, respectively. Moreover, Figure 3(b) further revealed the Kaplan-Meier OS curves of patients had stratified according to the ALBI grade. Patients with ALBI grade 3 had significantly worse OS compared with patients with ALBI grades 1–2 (*P* = 0.012). Accordingly, the 1-year OS rates of patients with ALBI grade 3 and ALBI grades 1–2 were 51.6% and 63.8%, respectively.

Subsequently, the prognostic significance of ALBI grade was analyzed in different subgroups based on the location of the lesion. As shown in Figures 3(c) and 3(d), ALBI grade was a significant predictor of OS both in the HCCA and in the DCCA subgroups (both *P* < 0.05).

The Kaplan-Meier curve in Supplementary Figure 1 revealed the significant association between CP score and OS (*P* = 0.016). Patients with CP score of ≥8 had significantly worse OS compared with patients with lower CP score.

**Figure 4:**
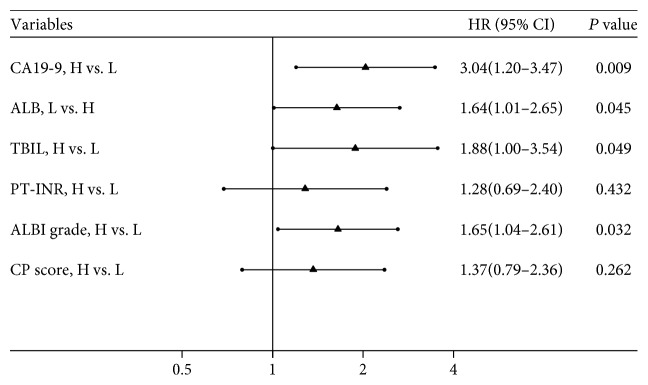
Forest plot based on the results of multivariate analysis of the factors associated with OS of EHC patients.

Univariate analysis demonstrated that preoperative TBIL, CA19-9, ALB, ALBI, and CP score were significant factors associated with OS (Table 2). PT-INR was nearly statistically significant (*P* < 0.10). Therefore, TBIL, CA19-9, ALB, and PT-INR entered into the multivariate analysis model, and CA19-9 (HR = 3.04, 95% CI: 1.20–3.47, *P* = 0.009), ALB (HR = 1.64, 95% CI: 1.01–2.65, *P* = 0.045), and TBIL (HR = 1.88, 95% CI: 1.00–3.54, *P* = 0.049) were found to be independent prognostic factors. Subsequently, CA19-9, PT-INR, and ALBI grade (or CP score) entered into the multivariate analysis model, and the result showed that ALBI (HR = 1.65, 95% CI: 1.04–2.61, *P* = 0.03), but not CP score (HR = 1.37, 95% CI: 0.79–2.36, *P* = 0.262), was independently associated with OS. The results of multivariate analysis were presented in the forest plot (Figure 4).

## 4. Discussion

EHC is a devastating malignancy with a poor outcome. Previous studies on potential prognostic factors of EHC are limited and unsatisfactory. Amongst preoperative ALB, TBIL, CP grade, tumor characteristics, and tumor markers such as CA19-9 and CEA have been established as important prognostic factors [18, 19]. However, the majority of the studies have assessed the prognostic factors only in EHC patients with surgical resection, while most EHC patients present with advanced stage at diagnosis and no curative treatments can be applied [4].

An increasing number of evidence has emphasized that liver function plays an important role in the progression of several types of solid tumors, including HCC, gastric cancer, colorectal cancer, and biliary tract neoplasms [8, 9, 15, 20]. Several scoring models are available to assess the severity of liver dysfunction as well as to predict the prognosis of patients with liver diseases or malignancies [7, 14, 15]. Presently, CP grade, which contains five parameters, including TBIL, serum ALB, PT, ascites status, and hepatic encephalopathy, is the most common model used in clinical practice. Recent studies have also shown that CP grade is an independent prognostic model in biliary tract neoplasms [8, 9, 20]. However, the highly subjective judgment of ascites and hepatic encephalopathy might reduce the accuracy of liver function assessment [21]. Ascites and encephalopathy may be due to tumor metastasis and cholangitis, but not liver dysfunction, in advanced EHC. Another two variables, ALB and ascites, are interactional and thus, the classification of ALB and ascites together may be redundant [22]. In addition, the majority of cases with EHC belong to high CP grade (97% cases were CP grade B in our study) as the high incidence of obstructive jaundice. Thus, the application of CP grade in EHC may not be appropriate.

In a systematic review of prognostic indicators of liver function, serum ALB and TBIL were two most prominent variables [23]. Recently, ALBI grade, which is simply calculated using only serum ALB and TBIL, is a newly emerging alternative of traditional CP grade for assessing the severity of liver dysfunction [24]. ALBI grade offers better performance of evaluating hepatic functional reserve and prognosis than CP grade in HCC [14, 15]. In our study, we firstly assessed the ALBI grade in advanced EHC patients. We found that, compared with the CP score, the ALBI score showed a higher degree of accuracy in predicting death. In addition, ALBI grade was found to be an independent prognostic model for OS.

The underlying mechanisms enabling the higher ALBI grade to indicate worse outcome in EHC are not well established. Firstly, liver dysfunction has been shown to be independently associated with hepatitis, liver cirrhosis, and biliary obstruction [25]—all of which increase the incident risk of EHC [26]. It is reported that approximately 70% of EHC cases have abnormal liver function [27]. Therefore, higher ALBI reflects liver dysfunction and results in poor prognosis in EHC [19]. Secondly, as shown in our study, higher ALBI grade is associated with higher CA19-9 and CRP, both of which are crucial prognostic factors of EHC [18, 28]. Thirdly, both ALB and TBIL in the ALBI grade contribute to the development and prognosis of various cancers, including EHC.

Hypoalbuminemia is associated with the progress of a variety of diseases. In several types of cancer, such as colorectal cancer, pancreatic carcinoma, hepatocellular carcinoma, and lung cancer, low ALB has broadly been recognized to be a poor prognostic indicator of survival [29]. Our previous study suggested that low ALB was associated with poor OS in patients with recurrent malignant obstructive jaundice [11]. Recently, low serum level of ALB has also been identified as an independent predictor of survival in EHC [19]. The potential mechanism may be multifactorial. Hypoalbuminemia is a frequent condition in patients with malnutrition and cachexia and increases the risk of antitumor drug-induced toxicity. In addition, hypoalbuminemia is associated with failure of various immune system components and promotes tumor growth.

Bilirubin is also essential for liver function and high level of serum TBIL is reflective of liver injury due to an injurious effect on hepatocytes. The majority of patients with EHC have elevated serum TBIL, as the lesion is located in the common bile duct and biliary confluence [11, 12]. Recent studies show that the high level of TBIL correlates with the development of EHC [30] and is an independent poor prognostic factor of EHC [31].

There are several limitations in the present study. Firstly, because of specific study population of advanced EHC, relatively small sample size, and single-center retrospective design, larger multicenter prospective studies are required to validate our findings. Secondly, the ALBI score was not dynamically measured after the operation. Whether postoperative ALBI grade or dynamic change of the ALBI score is associated with the prognosis of EHC remains unclear. Thirdly, ROC curve, Kaplan-Meier curve, and Cox regression were used to compare ALBI and CP scores, which are indirect methods of assessing OS. In contrast, time-dependent ROC is a direct method and may be more appropriate to compare the two staging systems.

## 5. Conclusion

In conclusion, for patients with advanced EHC who received PTBS combined with ^125^I seed implantation, ALBI grade may be a simple and valuable prognostic model. Whether our findings would be applicable to early EHC patients or advanced EHC patients who received other therapies needs to be further investigated.

## Figures and Tables

**Table 1:** Basic characteristics of the included patients.

Variables	Overall	ALBI grade		
		1-2 (*n* = 47)	3 (*n* = 62)	*P*
Gender (male/female)	71/38	28/19	43/19	0.289
Age	68.9 ± 11.1	67.2 ± 11.8	70.1 ± 10.4	0.155
Age (≥70/<70 years)	57/52	23/24	34/28	0.541
Location (HCCA/DCCA)	70/39	31/16	39/23	0.742
CA19-9 (ng/ml)	534.3 (0.6–2068)	291.3 (0.6–2068)	936.2 (0.6–1967)	**0.006**
CEA (ng/ml)	4.4 (0.9–310.4)	4.0 (0.9–294.8	5.5 (0.9–310.4)	0.432
ALT (U/l)	149 (22–531)	128 (24–375)	202 (22–513)	**0.006**
AST (U/l)	158 (28–541)	152 (28–541)	159.5 (35–365)	0.976
ALP (U/l)	553 (39–2556)	487 (39–2451)	646.5 (202–2556)	0.110
GGT (U/l)	543 (4.1–2528)	636 (69–2528)	492 (4.1–1706)	0.073
TBIL (*μ*mol/l)	249.7 (14.1–706.2)	161.8 (14.1–325.4)	306.9 (47.3–706.2)	**<0.001**
DBIL (*μ*mol/l)	201.4 (5.0–649.0)	146.9 (5.0–299.0)	261.4 (23–649	**<0.001**
ALB (g/l)	34.2 (23.6–45.3)	38.3 (33.1–45.3)	31.9 (23.6–35.7)	**<0.001**
CRP (mg/l)	16.8 (0.6–205.6)	11.7 (0.6–111.9)	28.2 (1.9–205.6)	**<0.001**
PLT (10^9^/l)	237 (54–533)	240 (117–483)	237 (54–533)	0.869
PT (s)	11.6 (9.3–16.5)	10.9 (9.4–12.9)	12.1 (9.3–16.5)	**<0.001**
PT-INR	1.02 (0.65–16.5)	0.98 (0.65–12.6)	1.07 (0.83–16.5)	**<0.001**
CP score 5/7/8/9/10	1/46/50/10/2	1/43/2/0/1	0/3/48/10/1	**<0.001**
Outcome (death/no)	86/23	30/17	55/7	**0.002**

ALBI: albumin-bilirubin; HCCA: hilar cholangiocarcinoma; DCCA: distal cholangiocarcinoma; CA19-9: carbohydrate antigen 19-9; CEA: carcinoembryonic antigen; ALT: alanine aminotransferase; AST: aspartate aminotransferase; ALP: alkaline phosphatase; GGT: gamma-glutamyl transpeptidase; TBIL: total bilirubin; DBIL: direct bilirubin; CRP: C-reactive protein; PLT: platelet count; ALB: albumin; PT: prothrombin time; PT-INR: prothrombin time-international normalized ratio; CP: Child-Pugh.

**Table 2:** Univariate analysis of factors associated with OS of EHC patients.

Variables	HR (95% CI)	*P*
Gender (male vs. female)	1.041 (0.663–1.636)	0.861
Age (≥70 vs. <70 years)	1.096 (0.712–1.687)	0.678
Location (HCCA vs. DCCA)	1.286 (0.806–2.054)	0.291
CA19-9 (≥643 vs. <643 ng/ml)	2.199 (1.376–3.516)	0.001
CEA (≥5.3 vs. <5.3 ng/ml)	1.460 (0.925–2.304)	0.104
ALT (≥113 vs. <113 U/l)	1.292 (0.805–2.075)	0.288
AST (≥102 vs. <102 U/l)	1.182 (0.679–2.058)	0.555
ALP (≥489 vs. <489 U/l)	1.247 (0.797–1.950)	0.334
GGT (≥158 vs. <158 U/l)	1.158 (0.595–2.256)	0.666
TBIL (≥162 vs. <162 *μ*mol/l)	2.334 (1.332–4.087)	0.003
ALB (≤35 vs. >35 g/l)	1.722 (1.097–2.703)	0.018
CRP (≥13.9 vs. <13.9 mg/l)	1.280 (0.818–2.001)	0.280
PLT (≥175 vs. <175 × 10^9^/l)	1.186 (0.628–2.239)	0.599
PT-INR (≥1.17 vs. <1.17)	1.647 (0.949–2.858)	0.076
ALBI grade (3 vs. 1–2)	1.724 (1.100–2.700)	0.017
CP score (≥8 vs. <8)	1.683 (1.076–2.633)	0.023

HCCA: hilar cholangiocarcinoma; DCCA: distal cholangiocarcinoma; CA19-9: carbohydrate antigen 19-9; CEA: carcinoembryonic antigen; ALT: alanine aminotransferase; AST: aspartate aminotransferase; ALP: alkaline phosphatase; GGT: gamma-glutamyl transpeptidase; TBIL: total bilirubin; CRP: C-reactive protein; PLT: platelet count; ALB albumin; PT-INR: prothrombin time-international normalized ratio; ALBI: albumin-bilirubin.

## Data Availability

The datasets used to support the findings of this study are available from the corresponding author upon request.

## Supplementary Materials

Supplementary Materials Supplementary Figure 1: The survival curve of unresectable EHC patients stratified according to CP score.
