# Supplementary material for: Albumin-Bilirubin Grade as a Novel Predictor of Survival in Advanced Extrahepatic Cholangiocarcinoma
Source: Gastroenterol Res Pract. 2018 Dec 2;2018:8902146. doi: 10.1155/2018/8902146 (PMC6304808; doi:10.1155/2018/8902146)
Supplement: Supplementary Materials — Supplementary Figure 1: The survival curve of unresectable EHC patients stratified according to CP score. [file 8902146.f1.docx]

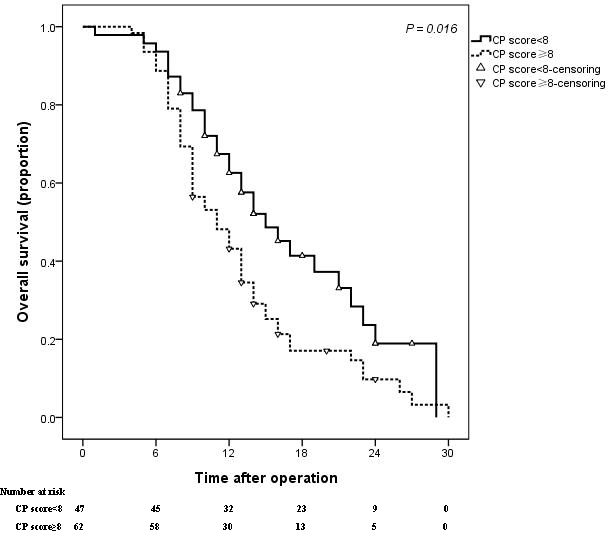


Supplementary FIGURE 1: The survival curve of unresectable EHC patients stratified according to CP score.
